# Factors associated with late diagnosis of breast cancer in women in Togo, Sub-Saharan Africa

**DOI:** 10.1186/s12905-023-02257-8

**Published:** 2023-03-14

**Authors:** Tchin Darré, Lantam Tchandikou, Panakinao Simgban, Mayi Bombone, Toukilnan Djiwa, Bidamin N’Timon, Bagassam Sama, Ayoko Ketevi, Baguilane Douaguibe, Bingo K. N’Bortche, Yao Seddoh, Mazamaesso Tchaou, Gado Napo-Koura

**Affiliations:** 1 Department of Pathology, University Teaching Hospital of Lomé, Lomé, Togo; 2 Faculty of Health Sciences, University of Lomé, BP 1515, Lomé, Togo; 3 Department of Imaging, University Teaching Hospital of Lomé and Kara, Lomé, Togo; 4 Department Obstetrics and Gynecology, University Teaching Hospital of Lomé, Lomé, Togo

**Keywords:** Breast, Cancer, Late diagnosis, Factors, Togo

## Abstract

**Background:**

Breast cancer is the most frequently diagnosed cancer in women worldwide. The objective of this study was to identify factors associated with late diagnosis of breast cancer in Togolese women.

**Methods:**

We conducted a prospective cross-sectional study with descriptive and analytical purposes on cases of breast cancer in women in 2021, in Togo. The patients included in this study were women followed in the gynecology department for stages III and IV breast cancer.

**Results:**

We included 62 cases of breast cancer. The average age of the patients was 38.6 ± 12.5 years with extremes of 17 and 76 years. The breast nodule was the most common reason for consultation in 75.8% of cases. The histological types diagnosed were invasive carcinoma of non-specific type (58; 93.55%), mucinous carcinoma (3; 4.84%) and lobular carcinoma (1; 1.61%). For the stage of the cancer, 43 patients were stage III (69.4%) and 19 stage IV (30.6%).

In multivariate analysis, the factors associated with late diagnosis of breast cancer were: fear of diagnosis (aOR = 1.29; *p* = 0.0014), long delay in diagnosis (aOR = 2.62; *p* = 0.0001) and failure to perform breast self-examination (aOR = 1.68; *p* = 0.0022).

**Conclusion:**

The fear of the diagnosis, the absence of self-examination of the breasts and the use of traditional treatment and self-medication in first intention constituted the essential factors of the late diagnosis of breast cancer. Strategies should be put in place at the national level to impact on these factors for an early diagnosis of breast cancer.

## Background

Breast cancer is the most common cancer worldwide, ahead of lung cancer with 2,261,419 new cases in 2020, or 11.7% of all cancer cases and 684,996 deaths, or 6.9% [1, 2]. During the same year, Africa recorded 1,109,209 cases including 49,339 cases in West Africa for 25,626 deaths [2]. Its incidence and mortality rates are expected to increase significantly over the next few years [3, 4]. Breast cancer incidence has increased significantly over the past two decades to 2.0% per year and is expected to reach over 19.3 million women by 2025, with the majority originating from sub-Saharan Africa [5]. In developing countries including Togo, breast cancer is a major public health issue, it is the leading cause of death among women with 324,000 deaths, which represents 14.3% of all deaths [6–8].

In Togo, a study carried out in 2016 reveals that breast cancer is the most frequently diagnosed in women with a prevalence of 21.2% of cancers in women and 10% of all cancers diagnosed in the country [9]. More recent data in 2020 show that this cancer is diagnosed at advanced stage III using the Nottingham classification (55.10%) [10]. In addition, more than half of the patients (52.1%) had T3-T4 tumors and the histoprognostic evaluation showed that grade 2 tumors were predominant (51.3%) followed by grade 3 (42.7%) [11–13]. Given the late diagnosis of breast cancer, the narrowness of the technical platform and the low standard of living of the population, we initiated this study, the aim of which was to identify factors associated with the late diagnosis of breast cancer in women in Togo.

## Methods

We report the results of a prospective cross-sectional study with descriptive and analytical purposes on cases of breast cancer in women admited and followed up in the gynecology department and confirmed histologically at the Laboratory of Pathological Anatomy and Cytology of the CHU Sylvanus Olympia in 2021. Togo is a country of 56,600 Km2, with an estimated population of 7,200,000 inhabitants, located between Ghana in the west, Benin in the east and Burkina faso in th north. The data were collected during an interview during the consultation with a histopathological confirmation of breast cancer at the Laboratory of Pathological Anatomy and Cytology from the registers of this laboratory. The patients included in this study were women followed in the gynecology department for stages III and IV breast cancer.

The parameters studied were demographic data (age, profession, parity, religious denomination, level of education and marital status); clinical date (reason for consultation, clinical size and topography); anatomopathological data (histological type, histoprognostic grade, pTNM stage as well as data relating to the diagnostic delay (accessibility to appropriate care structures, consultation time, fear of diagnosis, caregiver profile, delay in carrying out histology, the practice of early detection methods) [14]. About the profession, the informal sector was all women who carry out an activity of income but not declared and a civil servant when the work is formally declared.

A univariate and multivariate logistic regression was carried out in order to find the factors associated with the long delay in consultation. The independent variable was the long consultation delay coded 1 if yes and 0 if not. The long consultation period is mentioned if the period between the first symptom and the first consultation is greater than 6 months [4]. When the independent variable was statistically associated with the dependent variable during the univariate analysis with a degree of significance *p* ˂0.20, it was introduced into the initial model. The top-down step-by-step procedure was used for final model selection. It consisted of including the variables chosen in the initial model. Univariate analysis was used to estimate the odds ratio (OR) and its 95% confidence interval. The multivariate analysis made it possible to estimate the adjusted odds ratio (RCa) and its 95% confidence interval for each variable retained.

## Results

### Socio-demographic characteristics of patients

Table 1 summarizes the socio-demographic characteristics of the patients. We collected 62 cases of breast cancer. The average age of the patients was 38.6 ± 12.5 years with extremes of 17 and 76 years. All social strata were represented but the informal sector was the majority with 52 cases (83.9%), followed by civil servants with a percentage of 11.3%. Only civil servants had health coverage. According to the level of education, the patients were mostly college level. Married women represented 54.8%, followed by single women (25.8%). Patients who had children represented 47 cases (75.8) and 34 of them had at least 3 children.

**Table 1:** Socio-demographic characteristics of patients

	**Number (*N* = 62)**	**%**
**Age (years)**		
([15–25[)	3	4.8
([25–35[)	6	9.7
([35–45[)	13	21.0
([45–55[)	19	30.6
([55–65[)	16	25.8
([65–75[)	4	6.5
([75–85[)	1	1.6
**Profession**		
Informal sector	52	83.9
Official	7	11.3
Retired	2	3.2
Student	1	1.6
**Level of education**		
No	13	21
Primary	13	21
College	17	27.4
High school	5	8
University	14	22.6
**Martal status**		
Bride	34	54.8
Single	16	25.8
Widow	6	9.7
Concubinage	4	6.5
Divorced	2	3.2

### Clinical and mammographic characteristics of patients

**Fig. 1:**
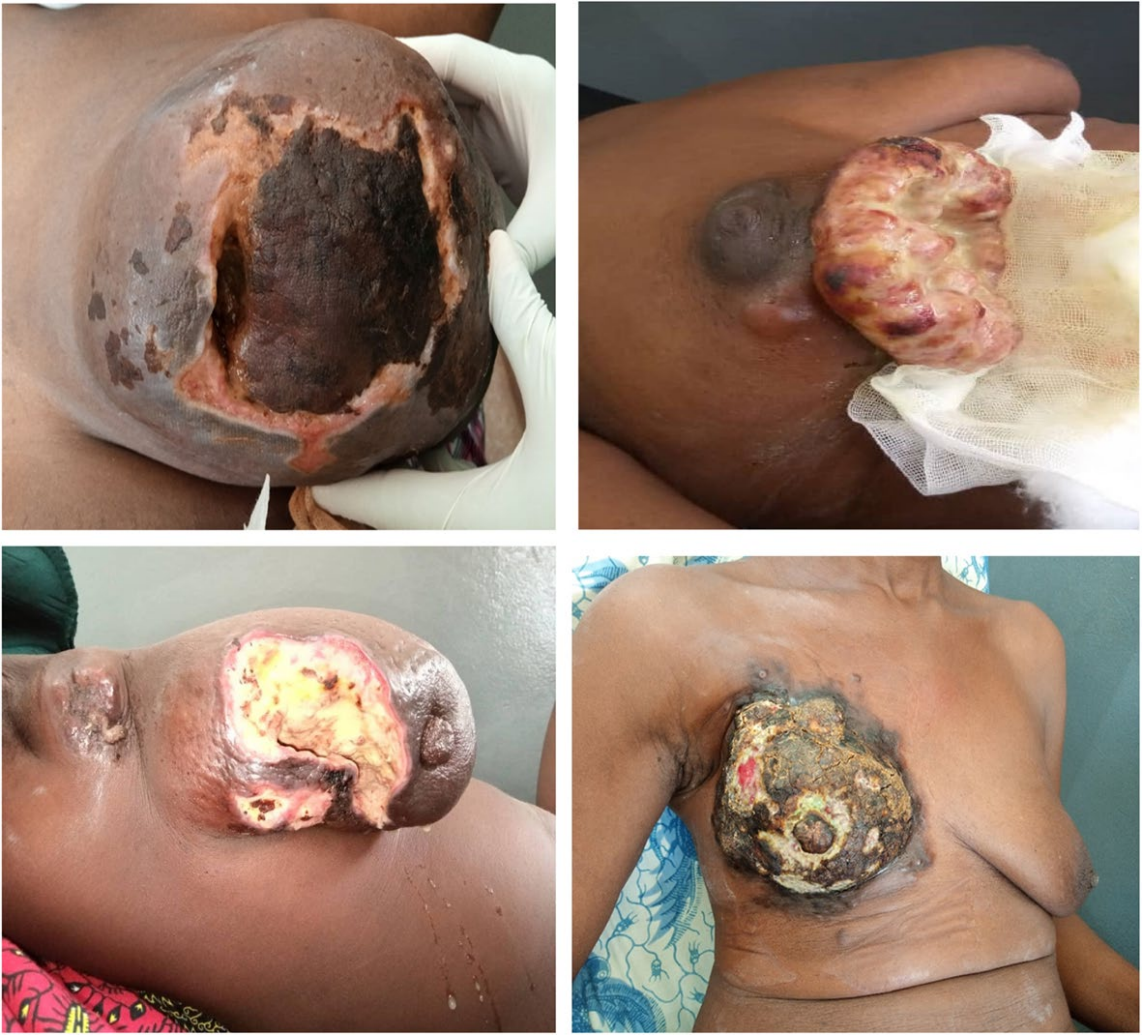
Clinical aspects of breast cancer patients

The breast nodule was the most common reason for consultation with 75.8% followed by breast swelling with 30.6%. The other reasons for consultation were mastodynia (29%), nipple discharge (11.3%), breast ulceration (11.3%) and abscess (1.6%) (Fig. 1). The left breast was the most affected in most women in our study with a percentage of 51.6%. The mean tumor size on mamography was 36.6 ± 50.1 mm with extremes of 17 mm and 240 mm (Table 2).

**Table 2:** Distribution of medical characteristics of the study population

	**Number (*N* = 62)**	**%**
**Topography**		
Left	32	51.6
Right	30	48.4
**Discovery mode**		
Breast nodule	47	75.8
Breast swelling	19	30.6
Mastodyna	18	29
Ulceration	7	11.3
Nipple discharge	7	11.3
Abcedure	1	1.6
**Histological type**		
Non specific infiltrating carcinoma	58	93.6
Mucinous carcinoma	3	4.8
Ivasive lobular arcinoma	1	1.6
**Histopronostic grade of Nottingham**		
I	7	11.3
II	45	72.5
III	4	6.5
Not specified	6	9.7
**Stade at diagnosis**		
III	43	69.4
IV	19	30.6

### Histopathological data

These were mastectomy pieces with axillary dissection. On section, the tumor presented a size varying between 3 and 11 cm long axis, with hemorrhagic and necrotic changes.The histological types diagnosed were invasive carcinoma of non-specific type (58; 93.55%), mucinous carcinoma (3; 4.84%) and lobular carcinoma (1; 1.61%). For the stage of the cancer, 43 patients were stage III (69.4%) and 19 stage IV (30.6%). The Nottingham histoprognostic grade was specified in 56 patients including 45 grade II, 7 grade I and 4 grade III.

### Factors studied in the context of delayed diagnosis

Thirty-seven patients (59.7%) claimed to have traveled less than 10 km to get to the care structure, 17 patients between 10 and 20 km and 8 patients more than 20 km. Breast self-examination was practiced by only 15 (24.2%) patients. The average consultation time was 23.6 ± 46.1 weeks with the extremes of one week and 288 weeks. The first consultation was with a doctor in 45 cases, a traditional healer in 9 cases. Eight patients had taken self-medication before the medical consultation. The average time taken to honor a histological examination was 5.7 ± 9.3 months with extremes of one day to 49.7 months. The average time taken from the first symptoms to the diagnosis of breast cancer was 6.3 ± 10.2 months with the extremes of 8 days and 50.07 months.

In univariate analysis, the factors associated with late diagnosis of breast cancer were: distance from health care structures with (*p* = 0.0037), fear of diagnosis (*p* = 0.00001), type of first consultation (*p* = 0.0026) andabsence of practice of self-examination of the breasts (*p* = 0.0012) (Table 3). In multivariate analysis, the factors associated with late diagnosis of breast cancer were: fear of diagnosis (aOR = 1.29; *p* = 0.0014), long delay in diagnosis (aOR = 2.62; *p* = 0.0001) and failure to perform breast self-examination (aOR = 1.68; *p* = 0.0022) (Table 4).

**Table 3:** Univariate analysis of the different factors involved in the late diagnosis of breast cancer in women in Togo

Factors	Diagnostic time			
	** < 6 months (35)** **n(%)**	** ≥ 6 months (27)** **n(%)**	**Total (62)** **n(%)**	***p*-value**
**Distance from health care facilities**				**0.0037**
No	30(85.7)	24(88.9)	54(87.1)	
Yes	5(14.3)	3(11.1)	8(12.9)	
**Fear of diagnosis**				**0.00001**
No	34(97.1)	26(96.3)	60(96.8)	
Yes	1(2.9)	1(3.7)	2(3.2)	
**Diagnostic error**				0.187
No	32(91.4)	27(100)	59(95.2)	
Yes	3(8.6)	0(0)	3(4.8)	
**Lack of financial means**				0.0851
No	33(94.3)	27(100)	60(96.8)	
Yes	2(5.7)	0(0)	2(3.2)	
**Type of first consultation**				**0.0026**
Physician	29(82.9)	16(59.3)	45(72.6)	
Traditherapeute	2(5.7)	7(25.9)	9(14.5)	
Self-medication	4(11.4)	4(14.8)	8(12.9)	
**Breast self-examination**				**0.0012**
No	22(62.9)	25(92.6)	47(75.8)	
Yes	13(37.1)	2(7.4)	15(24.2)

**Table 4:** Multivariate analysis of the different factors involved in the late diagnosis of breast cancer in women in Togo

**Factors**			**Univariate analysis**			**Multivariate analysis**		
			**Long consultation time**			**Long consultation time**		
	**n/N**	**%**	**aOR**	**IC95%**	***P*-value**	**aOR**	**IC95%**	***p*-value**
**Distance from health care facilities**					0.076			
No (54)	24/54	44.4	1	-				
Yes (8)	3/8	38	0.78	1.33–2.5				
**Fear of diagnosis**					**0.018**			**0.0014**
No (60)	26/60	43.3	1	-		1	-	
Yes (2)	1/2	50	1.18	1.09–2.32		1.29	1.36–2.58	
**Diagnostic error**					1.722			
No (59)	27/59	45.8	1.56	1.26–4.28				
Yes (3)	0/3	0	1	-				
**Lack of financial means**					0.91			
No (60)	27/60	45	2.2	1.23–3.45				
Yes (2)	0/2	0	1	-				
**Type of first consultation**					**0.0025**			**0.0001**
Physician (45)	16/45	35.6	1	-		1	-	
Traditherapeute (9)	7/9	77.8	2.15	1.06–4.4		2.62	2.01–4.7	
Self-medication (8)	4/8	50	1.52	1.18–2.24		1.29	1.07–2.78	
**Breast self-examination**					**0.0062**			**0.0022**
No (47)	25/47	53.2	1.54	1.13–3.1		1.68	1.47–2.27	
Yes (15)	2/15	13.3	1	-		1	-	

## Discussion

Late diagnosis of breast cancer remains a topical problem in our practice environment. In our series, as in most African publications, the diagnostic delay was particularly long, in our series the average delay is 6.3 months [15–17]. The reasons for the late diagnosis were the remoteness of health facilities, the fear of the diagnosis, the type of first consultation, the lack of practice of self-examination of the breasts. Socio-cultural habits represented by first-line use of traditional medicine and the problem of staff qualification were also reasons for late diagnosis of breast cancer [11, 13]. The training of nursing staff, especially general practitioners, nurses and midwives, should be able to limit the risk of diagnostic errors insofar as breast cancer often poses a problem of differential diagnosis with benign mastopathies [18–21].

The first factor obtained was fear of the diagnosis (*p*-value = 0.0014; RCa = 1.29; 95% CI [1.36–2.58]). Half of these patients had consulted after a period of six months against 43.3% of patients who had no phobia related to this condition. Thus, for a woman, being afraid of being diagnosed with breast cancer would increase the risk of diagnosing it at the late stage by 1.29% [2, 6]. The patient's psychic experience is heavily affected, with the psychological disorders that usually accompany the announcement of any cancer, passing through the acceptance of the disease until mourning, such as: depression, anxiety and fear of death, will be grafted other disorders, which can be as reprimanding as the disease itself [22].

The second factor was the type of consultation opted for the detection of symptoms (with a *p*-value = 0.0001). Some patients opted for traditional therapy, 77.8% had a long delay in diagnosis (RCa = 2.62; 95% CI [2.01–4.7]). Similarly, half of those who had chosen the self-medication route had a long diagnostic delay (RCa = 1.29; 95% CI [1.07–2.78]). This means that moving towards traditional treatment would increase the chance of being diagnosed with breast cancer at an already advanced stage by 2.62%, while self-medication would increase it by 1.29%. The circuit of patients suffering from breast cancer is often complex in Africa. Most turn to traditional healers who waste patients' time both in diagnosis and in therapeutic management. This would partly explain the fact that cancer patients often arrive late in health structures and often with complications or even metastases [23]. Beliefs and especially ignorance would be the parameters that direct patients to these traditional healers who unfortunately do not know their limits in the management of serious and chronic pathologies such as cancer [24].

The three factor was failure to perform breast self-examination. Al-though self-examination is the most commonly used early detection technique,evidence of its effectiveness is discussed [11, 25]. This technique can lead toover detection of nodules leading to unnecessary visits to the doctor and ex-penses related to the diagnosis, which is not desirable in the context of development countries where health facilities and resources are limited [26]. However, al-though current evidence does not support self-examination as a breast cancerscreening approach, teaching breast self-examination at the individual level incountries where most women with breast cancer advanced disease may improveawareness of breast cancer and lead to an earlier stage of diagnosis [27, 28]. Clinicalbreast examination has the advantage of being a relatively simple and inexpen-sive technique for the early detection of breast tumors [10]. It should be doneadequately by trained health workers [28, 29]. The role of self-examination andclinical breast examination is important in areas where mammography may notbe available for financial and accessibility reasons [29]. In addition, statistics in-dicate that 90% of breast nodules are discovered by women themselves [29]. As for the students’ knowledge of mammography as a means of screening forbreast cancer, 75.3% were aware of it. Mammographic screening proves to be beneficial when it is car-ried out in an organized and regular manner in the form of a national publichealth policy or when the per capita income of the population allows it to absorbmost of the costs [30]. Mammography screening is financially and technically difficult to implement and maintain, requiring high-quality machines, well-trainedradiologists and technicians, and investment in pathology and treatment facili-ties [31]. Therefore, organized screening is difficult in developing countries. Socioeconomic dependence and late diagnosis reflect higher rates of breast can-cer morbidity and mortality in developing countries [17, 29–31]. The impact of mam-mography screening may be more beneficial in developing countries than whathas been observed in developed countries [17].

A percentage of 53.2% of this group had consulted after six months (RCa = 1.68; 95% CI [1.47–2.27]), which means that a woman not practicing breast self-examination would have a 1.68% chance of being diagnosed with late-stage breast cancer [22, 32]. The psychic experience of the patient is heavily affected. The psychological disorders that usually accompany any announcement of cancer, ranging from acceptance of the disease to mourning; such as depression, anxiety and fear of death [1, 18]. Other disorders will be added, in particular the devaluation or loss of self-esteem, isolation, disorders of body image and/or sexual identity, which can be as reprimanding as the disease itself [4, 19]. The social experience of the patient who will continually avoid the gaze of others is another severe test [22, 32]. Several of these incriminated factors, taken individually, have an impact on the long diagnostic delay [2–5, 33, 34]. This is the case of the time taken to carry out the anatomopathological examination. Indeed, to date, Togo only has one pathological anatomy and cytology laboratory; which considerably increases the delay in histological diagnosis [7–9]. On the clinical level, the questioning in our series showed that less than 24.2% of the patients practiced self-examination which represents an important act in the early diagnosis of breast cancer. In the West, on the other hand, it constitutes, along with systematic screening, a habitual reflex in women, so that 80% of patients are seen at early stages [35–38]. The circumstances of discovery in our series were related to a pejorative stage and dominated by breast nodule with 75.8% followed by breast swelling with 30.6%. In developed countries, early diagnosis predominated at the stage of nodules and subclinical lesions detected by screening mammography [39–41].

## Conclusion

The fear of the diagnosis, the absence of self-examination of the breasts and the recourse to traditional treatment and self-medication in first intention constituted the essential factors of the late diagnosis of breast cancers. This fear is generated by the gloomy prognosis to which it is subject and the cost of its treatment. The fact that women do not systematically practice self-examination explains why this cancer is always diagnosed in its symptomatic phase. The use of parallel treatments lengthens the diagnostic delay and prolongs the evolution and development time of the disease. Screening goes through information, education and the fight against poverty. Early screening of breast cancer requires information to the population, education and the fight against poverty. This early screening should contribute to the fight against the problem of delayed diagnosis in our context.

## Data Availability

Extracted data are with the corresponding author and available under reasonable request.

## Abbreviations

AJCC: American Joint Committee on Cancer
TNM: Tumor, Nodes, metastasis
OR: Odds ratio
